# Endoscopic Reduction of an Acute Gastric Volvulus

**DOI:** 10.7759/cureus.58198

**Published:** 2024-04-13

**Authors:** Prisca Pungwe, Dirin Ukwade, Ankur Patel, Teminioluwa A Ajayi, Gyanprakash Ketwaroo

**Affiliations:** 1 Internal Medicine, Baylor College of Medicine, Houston, USA; 2 Internal Medicine, University of Illinois Chicago, Chicago, USA; 3 Gastroenterology and Hepatology, Baylor College of Medicine, Houston, USA

**Keywords:** endoscopic decompression, paraesophageal hernia, gastric volvulus, endoscopic detorsion, acute volvulus

## Abstract

We are reporting a case of gastric volvulus in a 52-year-old man in the setting of a paraesophageal hernia initially identified on computerized tomography (CT). CT of the abdomen showed a large paraesophageal hernia with intra-thoracic herniation of the distal stomach and gastroduodenal junction, resulting in mesenteroaxial rotation consistent with acute gastric volvulus. Esophagogastroduodenoscopy (EGD) confirmed the presence of the gastric volvulus, which was initially temporized with endoscopic detorsion. He subsequently had nasogastric tube placement and ultimately underwent a laparoscopic gastropexy. He recovered uneventfully with plans for Roux-en-Y gastric bypass surgery.

## Introduction

A gastric volvulus is a rare event that occurs when the stomach rotates onto itself along its transverse or longitudinal axis. Most patients present with mild or intermittent gastric obstructive symptoms [1]. However, some patients may present with more extreme abdominal pain indicative of gastric necrosis or ischemia [2,3]. The majority of cases are referred for emergent surgical repair. However, conservative management with endoscopic decompression followed by a surgical gastropexy has shown to be similarly efficacious [4,5]. We present a case of a 52-year-old man who underwent successful endoscopic reduction of a gastric volvulus with subsequent surgical gastropexy.

This article was previously presented as a poster at the 2023 American College of Gastroenterology meeting on October 22, 2023.

## Case presentation

**Figure 1:**
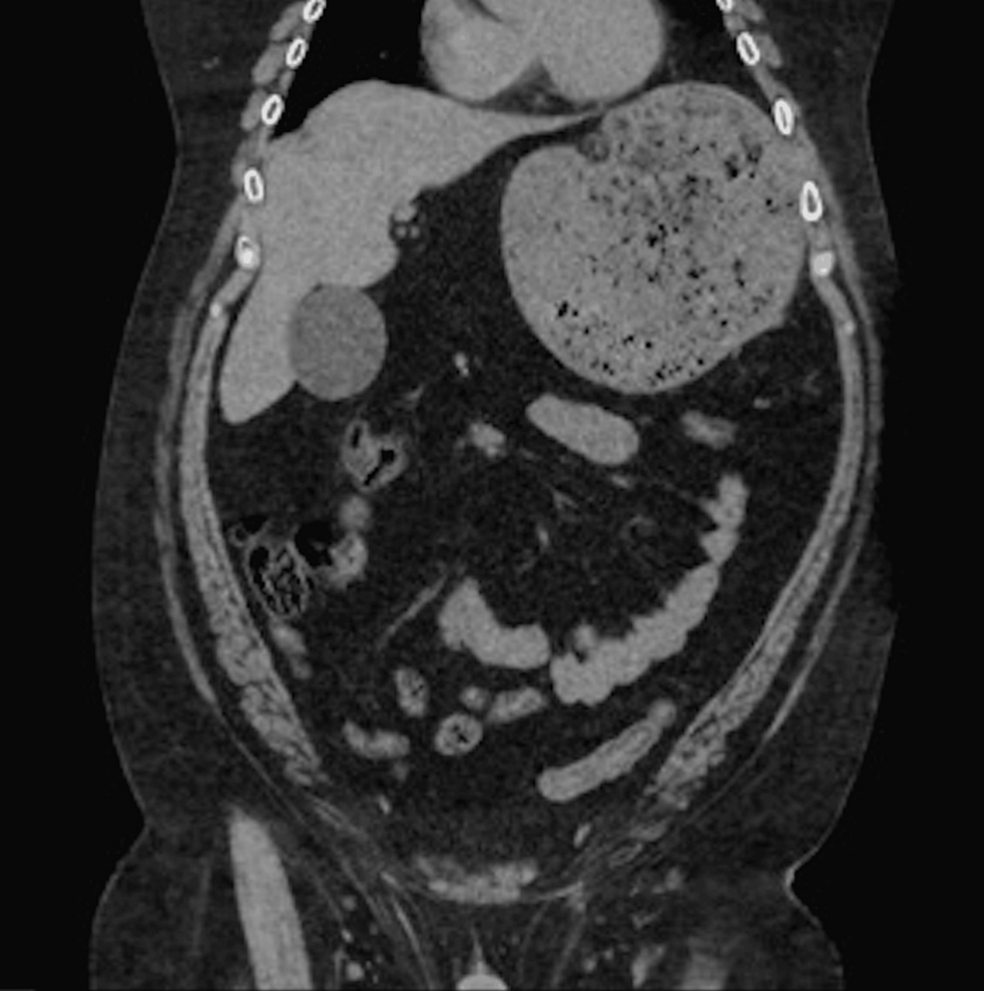
Coronal CT before decompression of gastric volvulus

A 52-year-old man with a history of morbid obesity and a paraesophageal hernia presented with one day of refractory epigastric pain and heartburn. Initial vital signs were notable for systolic blood pressure of 162 mmHg. A physical exam revealed tenderness in the mid-epigastric region. Computerized tomography (CT) of the abdomen showed a large paraesophageal hernia with intra-thoracic herniation of the distal stomach and gastroduodenal junction. Additionally, Mesenteroaxial rotation was present, consistent with acute gastric volvulus (Figure 1).

**Figure 2:**
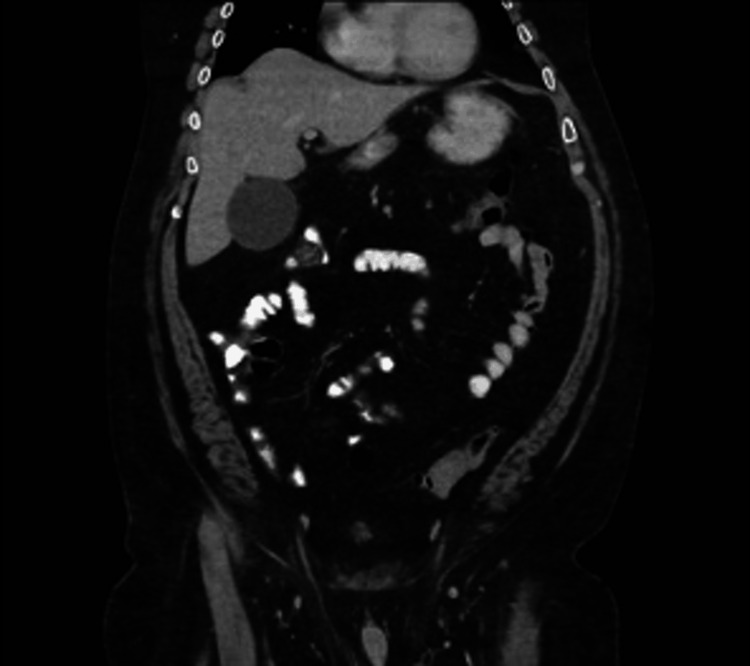
Coronal CT after decompression of gastric volvulus

After surgical consultation, an esophagogastroduodenoscopy (EGD) was performed, which showed torsion of the gastric body and antrum with intra-thoracic herniation and no evidence of mucosal ischemia. Following extensive suctioning of gastric debris, the herniated parts of the distal stomach were detorsed. Following detorsion, a nasogastric tube was placed for decompression. CT imaging after endoscopic intervention showed an improved reduction in bowel loops (Figure 2).

The patient subsequently underwent laparoscopic gastropexy and recovered uneventfully. He was discharged with a plan for definitive management with Roux-en-Y gastric bypass.

## Discussion

Although gastric volvulus is rare, presentations can range from mild obstructive symptoms to severe ischemia, sepsis, or gastric perforation. Gastric volvulus usually affects children less than one year and adults older than 50 [1,4]. Up to 30% of gastric volvulus cases are described as primary, from laxity and disruption of the stomach’s ligaments (gastrohepatic, gastrocolic, gastrolienal, and gastrophrenic) [1]. The other 70% of cases result from an anatomic issue involving the stomach, spleen, or diaphragm, including paraesophageal hernias [1].

Diaphragmatic hernias involve the migration of the gastroesophageal (GE) junction from the abdomen into the chest [3]. There are four types of diaphragmatic hernias. Type 1 diaphragmatic hernias are known as sliding hernias and represent 95% of all diaphragmatic hernias [2]. In type 1 sliding hernias, the GE junction slides above the diaphragm along with part of the cardia of the stomach [3]. Type 2-4 are known as paraesophageal hernias. These hernias occur when the GE junction herniates adjacent to or directly alongside structures such as the stomach. This is due to the weakening of the phreno-esophageal ligament [3]. In type 2 paraesophageal hernias, the GE junction remains at or below the level of the diaphragm. The resultant hernia forms due to the anteriorly displaced gastric fundus [2,3]. Type 3 paraesophageal hernias occur when the GE junction and gastric fundus herniate anteriorly together. Lastly, type 4 paraesophageal hernias occur when the GE junction, gastric fundus, and additional viscera all herniate together anteriorly [3]. Types 2-4 are rare but have a high risk of evolving into gastric volvulus [6]. Our patient likely had a type 3 or 4 paraesophageal hernia, given the possible herniation of the gastroduodenal junction.

Acute gastric volvulus secondary to paraesophageal hernia is frequently addressed with emergent surgery [5,6]. However, alternative approaches, including initial endoscopy followed by gastropexy and watchful waiting followed by elective surgery, have shown to be similarly efficacious [4,5]. Kaplan et al. compared emergency paraesophageal hernia repairs to elective paraesophageal hernia repairs, showing decreased mortality and hospitalization duration in those who underwent elective repairs [1]. Shea et al. also showed that complications occurred at a similar rate between emergent and elective paraesophageal hernia repairs, suggesting that outcomes would not be worse with watchful waiting than eventual repair [5]. Wirsching et al. further evaluated a staged approach for paraesophageal hernia repair, with endoscopic decompression followed by semi-elective surgery [7]. There was no difference in post-operative Clavien-Dindo severity scores between urgent surgery and staged procedural approaches [7]. Management of gastric volvulus with gastropexy has also shown to be successful. Yates et al. and Takahashi et al. evaluated laparoscopic gastropexy in patients with acute gastric volvulus, with successful outcomes [8,9].

## Conclusions

Ultimately, the method chosen for managing acute gastric volvulus depends on the initial clinical presentation. Patients who present with acute gastric volvulus without evidence of critical illness, such as gastric necrosis, ischemia, or sepsis, can be managed successfully with initial endoscopic intervention. These patients are also candidates to receive a gastropexy before eventual elective surgery. Our case adds to the present data for validating endoscopic reduction followed by surgical gastropexy as a viable method to approach paraesophageal hernias complicated by acute gastric volvulus.
